# Moderate to Severe Thrombocytopenia in Four Pregnant Women With Asymptomatic COVID-19 Infection

**DOI:** 10.7759/cureus.18531

**Published:** 2021-10-06

**Authors:** Sharad Kumar, Anisha Choudhary, Rajiv Shukla, Vinita Singh, Rajeev Ranjan

**Affiliations:** 1 Anesthesiology, Tata Main Hospital, Jamshedpur, IND; 2 Obstetrics and Gynecology, Tata Main Hospital, Jamshedpur, IND

**Keywords:** asymptomatic, covid-19, pregnancy, thrombocytopenia, coronavirus

## Abstract

Coronavirus disease 2019 (COVID-19) pandemic is one of the biggest healthcare crises faced globally. Since its emergence, uncertainty about its progress and treatment options has challenged clinicians around the world. Pregnant women infected with severe acute respiratory syndrome coronavirus 2 (SARS-CoV-2) infection pose a higher challenge due to the concerns of an already altered immune system during pregnancy and the disease's effect on the fetus. Thrombocytopenia is associated frequently with moderate to severe coronavirus disease and is also an established marker of worsening of the disease. However, it is infrequently seen in mild or asymptomatic cases.

Neuraxial anesthesia is the preferred choice of anesthesia in COVID-19 positive patients but thrombocytopenia in a parturient with coronavirus disease can cause a dilemma for the obstetric anesthesiologist. Here we describe the management of four pregnant women with asymptomatic COVID-19 disease who had moderate to severe thrombocytopenia. These cases highlight the importance of careful monitoring of the platelet count of pregnant women with COVID-19 infection even if asymptomatic.

## Introduction

The current coronavirus disease 2019 (COVID-19) pandemic continues to rage through the world mercilessly. Pregnant women belong to a vulnerable group of the population due to dual concerns about the health of the mother and the baby. Involvement of the respiratory system is commonly seen among the infected individuals; however, other system involvements like hematological, cardiac, gastrointestinal, hepatic, renal, neurological, and ocular are also being reported in rising numbers [1]. COVID-19 disease may manifest in varying degrees of severity ranging from asymptomatic presentation to moderate and severe disease. Current literature shows that pregnant women diagnosed with COVID-19 infection are less likely to manifest symptoms of fever, dyspnea, and myalgia than non-pregnant women of reproductive age; however, they are at increased risk of admission to intensive care unit (ICU) and receiving invasive ventilation [2].

Platelet count is a simple and easily available biomarker. Thrombocytopenia is seen in 5%-40% of COVID-19 infected non-pregnant patients [3]. A meta-analysis by Lippi et al. included nine studies in patients with COVID-19 disease and reported a significant association between thrombocytopenia at admission and increased risk of severe disease and mortality [4]. However, moderate to severe thrombocytopenia in asymptomatic COVID-19 patients is rarely reported and studies on pregnant women are scarce. Pregnant women with severe thrombocytopenia carry the risk of intrapartum and postpartum hemorrhage and a higher risk of epidural hematoma following neuraxial anesthesia. In this case series, we describe the management and outcome of four pregnant women with asymptomatic COVID-19 infection with moderate to severe thrombocytopenia.

## Case presentation

Case 1

A 30-year-old primigravida was admitted at 38 weeks of gestation with pain in the abdomen for two hours. On admission, she was afebrile, her pulse rate was 92 beats/minute, blood pressure was 112/74 mmHg, respiratory rate was 14 breaths/minute, and oxygen saturation was 99% in room air. As per institutional protocol, a rapid antigen test for COVID-19 was done, which was positive. The patient was shifted to the COVID-19 positive isolation ward. The patient had conceived spontaneously and had regular antenatal visits. She had no history of fever, cough, myalgia, or any contact history with a positive patient. She had no history of any past medical disorders. Her routine blood investigations were sent, which showed moderate thrombocytopenia (Table 1).

**Table 1:** Laboratory investigations of the patient on the day of admission. TLC: total leukocyte count; ALT: alanine transaminase; AST: aspartate aminotransferase; ALP: alkaline phosphatase; LDH: lactate dehydrogenase; CRP: C-reactive protein.

Parameter	On admission	Normal range
Hemoglobin (gm/dl)	10.1	11.5-16.5
TLC (cells per mm^3^)	7,400	4,000-11,000
Platelet (cells per mm^3^)	76,000	150,000-450,000
Prothrombin time (seconds)	10.8	11-16
International normalized ratio	1.01	0.8-1.2
Total bilirubin (mg/dl)	0.39	0.2-1
ALT (U/l)	27	5-40
AST (U/l)	47	5-45
ALP (U/l)	134	35-125
LDH (U/l)	120	208-378
Serum creatinine (mg/dl)	0.6	0.5-1.5
CRP (mg/L)	0.8	0-5

Her ultrasound showed a live fetus and examination revealed thick meconium-stained liquor. The patient had a cesarean section under spinal anesthesia. The cesarean section was uneventful, but the patient had postpartum hemorrhage due to uterine atony, which was managed with injection oxytocin, injection carboprost, and injection tranexamic acid. A repeat test was done which showed a hemoglobin level of 6.6 gm/dl and platelet count of 28,000. The patient received six units of random donor platelet concentrates and two units of packed red blood cells transfusion after which her hemoglobin level was 9.2 gm/dl and platelet count was 106,000 cells per mm^3^. The patient recovered well and did not develop any symptoms of COVID-19 disease on further follow-up.

Case 2

A 28-year-old primigravida was admitted at 38 weeks four days of gestation with complaints of pain in the abdomen and leaking per vagina for four hours. On examination, she was afebrile, her pulse rate was 98 beats/minute, blood pressure was 110/70 mmHg, and oxygen saturation was 98% in room air. She tested COVID-19 positive by reverse transcription-polymerase chain reaction (RTPCR) assay on a nasopharyngeal swab. The patient had regular antenatal visits and had no history of any medical disorder. She had no history of any symptoms suggestive of COVID-19 disease. Her routine blood investigations were sent, which were as follows (Table 2).

**Table 2:** Laboratory investigations of the patient on the day of admission. TLC: total leukocyte count; ALT: alanine transaminase; AST: aspartate aminotransferase; ALP: alkaline phosphatase; LDH: lactate dehydrogenase; CRP: C-reactive protein.

Parameter	On admission	Normal range
Hemoglobin (gm/dl)	10.1	11.5-16.5
TLC (cells per mm^3^)	5,700	4,000-11,000
Platelet (cells per mm^3^)	62,000	150,000-450,000
Prothrombin time (seconds)	10.9	11-16
International normalized ratio	1	0.8-1.2
Total bilirubin (mg/dl)	0.57	0.2-1
ALT (U/l)	17	5-40
AST (U/l)	37	5-45
ALP (U/l)	134	35-125
LDH (U/l)	197	208-378
Serum creatinine (mg/dl)	0.56	0.5-1.5
CRP (mg/L)	0.61	0-5

The patient was monitored for the progress of labor and delivered a live baby vaginally. She was monitored closely during the postpartum period and recovered well. She did not receive platelet transfusion and did not develop symptoms of COVID-19. She was followed up after 14 days and her platelet count had returned to normal range.

Case 3

A 28-year-old gravida 3 para 2 living 2 at eight weeks of gestation came to the emergency department with complaints of pain in the abdomen and vomiting. She was afebrile on admission, her pulse rate was 110 beats/minute, blood pressure was 100/70 mmHg, and oxygen saturation was 98% in room air. Her rapid antigen test for COVID-19 was positive and the patient was admitted to the COVID-19 positive isolation area. Her ultrasound revealed a hemoperitoneum with ruptured left tubal ectopic pregnancy. The patient was posted for laparotomy and her routine investigations were sent (Table 3).

**Table 3:** Laboratory investigations of the patient on the day of admission. TLC: total leukocyte count; ALT: alanine transaminase; AST: aspartate aminotransferase; ALP: alkaline phosphatase; LDH: lactate dehydrogenase; CRP: C-reactive protein.

Parameter	On admission	Normal range
Hemoglobin (gm/dl)	7.6	11.5-16.5
TLC (cells per mm^3^)	15,200	4,000-11,000
Platelet (cells per mm^3^)	70,000	150,000-450,000
Prothrombin time (seconds)	14.7	11-16
International normalized ratio	1.39	0.8-1.2
Total bilirubin (mg/dl)	0.28	0.2-1
ALT (U/l)	14.6	5-40
AST (U/l)	32	5-45
ALP (U/l)	57.5	35-125
LDH (U/l)	189	208-378
Serum creatinine (mg/dl)	0.56	0.5-1.5
CRP (mg/L)	5.7	0-5

After initial resuscitation, general anesthesia was induced. Laparotomy was done, and clots of approximately 1.5 liters of blood were removed. Left salpingectomy with right tubal ligation was done. The patient received two units of packed red blood cells, four units of random donor platelet concentrates, and six units of fresh frozen plasma intra-operatively and was shifted to the COVID-19 positive intensive care unit. She was extubated on second post-operative day, and her repeat investigation showed a hemoglobin level of 8.4 gm/dl, platelet count of 118,000 cells per mm^3^. On post-operative day three, the patient complained of breathlessness and her oxygen saturation dropped to 85% in room air. She was started on non-invasive ventilation to maintain optimum oxygen saturation level. Her repeat investigations were sent, which showed an increasing trend of C-reactive protein (CRP) and lactate dehydrogenase (LDH) levels (Table 4).

**Table 4:** Laboratory investigations of the patient on the days of admission. TLC: total leukocyte count; ALT: alanine transaminase; AST: aspartate aminotransferase; ALP: alkaline phosphatase; LDH: lactate dehydrogenase; CRP: C-reactive protein.

Parameter	Post-operative day 3	Before discharge	Normal range
Hemoglobin (gm/dl)	8.5	8.3	11.5-16.5
TLC (cells per mm^3^)	7,600	5,200	4,000-11,000
Platelet (cells per mm^3^)	120,000	132,000	150,000-450,000
Prothrombin time (seconds)	11.3	10.4	11-16
International normalized ratio	1.04	0.98	0.8-1.2
Total bilirubin (mg/dl)	0.45	0.6	0.2-1
ALT (U/l)	44.8	88	5-40
AST (U/l)	56	102	5-45
ALP (U/l)	67.5	145	35-125
LDH (U/l)	787	290	208-378
Serum creatinine (mg/dl)	0.68	0.66	0.5-1.5
CRP (mg/L)	22.8	1.4	0-5
D-Dimer (ng/ml)	3210	-	<500

In the background of continuous oxygen requirement and high D-dimer level, the patient was started on injection remdesivir, injection dexamethasone, and injection enoxaparin. She responded well to the treatment and her oxygen requirement started to decrease after three days and she was maintaining saturation of more than 95% in room air after five days. Remdesivir was stopped after five days and the patient was discharged on post-operative day 10 in a stable condition.

Case 4

A 28-year-old gravida 2 para 1 living 1 at 39 weeks four days of gestation was admitted with chief complaints of pain in the abdomen for two hours. The patient had tested COVID-19 positive two days earlier, was asymptomatic, and was in home isolation. On admission, the patient was afebrile, her pulse rate was 88 beats/minute, blood pressure was 112/78 mmHg, and oxygen saturation was 99% in room air. The patient was a known case of gestational diabetes mellitus and was on tablet metformin 500 mg twice daily. The patient was in early labor and her routine blood investigations were sent (Table 5).

**Table 5:** Laboratory investigations of the patient on the day of admission. TLC: total leukocyte count; ALT: alanine transaminase; AST: aspartate aminotransferase; ALP: alkaline phosphatase; LDH: lactate dehydrogenase; CRP: C-reactive protein.

Parameter	On admission	Normal range
Hemoglobin (gm/dl)	11.5	11.5-16.5
TLC (cells per mm^3^)	7,500	4,000-11,000
Platelet (cells per mm^3^)	91,000	150,000-450,000
Prothrombin time (seconds)	12.7	11-16
International normalized ratio	1.19	0.8-1.2
Total bilirubin (mg/dl)	0.48	0.2-1
ALT (U/l)	45	5-40
AST (U/l)	54	5-45
ALP (U/l)	188	35-125
LDH (U/l)	129	208-378
Serum creatinine (mg/dl)	0.65	0.5-1.5
CRP (mg/L)	0.28	0-5

Progress of labor was monitored, and the decision of cesarean section was taken in view of repeated late decelerations on electronic fetal monitoring. A repeat platelet count was done, which was 75,000 cells per mm^3^. In view of the rapidly declining platelet count, four units of random donor platelet concentrate transfusion were done. The patient had a cesarean section under spinal anesthesia. Intraoperative and postoperative period was uneventful. The patient was discharged after three days and the platelet count before discharge was 130,000 cells per mm^3^.

## Discussion

Gestational thrombocytopenia (GT) is defined as a platelet count below 150,000 cells per mm^3^ and occurs in approximately 5% to 10% of women during the third trimester of pregnancy or in the immediate postpartum period [5]. GT is usually an incidental finding, but it may also indicate coexisting systemic disorders like preeclampsia and hemolysis, elevated liver enzymes, and low platelet (HELLP) syndrome. In the majority of women, GT is mild, only 1%-5% develop platelet count below 100,000 cells per mm^3^, and very few have counts below 50,000 cells per mm^3^, which is not attributed to any other etiology.

Hematological changes like reduced lymphocyte and platelet count are frequently seen in patients infected with severe acute respiratory syndrome coronavirus 2 (SARS-CoV-2) infection. The possible mechanism is thought to be either direct infection of bone marrow cells by the virus, platelet aggregation and destruction by the immune system, and increased platelet consumption by microthrombi generation [6]. Studies in consecutive patients with COVID-19 have reported that thrombocytopenia (a platelet count of <150,000 cells per mm^3^) can be found in 70%-95% of patients with severe COVID-19 disease [7].

Three of our patients were in the third trimester but all our patients had a platelet count of less than 100,000 cells per mm^3^ and as low as 28,000 cells per mm^3^ in the first case, making the diagnosis of thrombocytopenia due to COVID-19 infection more likely. Preeclampsia and HELLP syndrome are also associated with thrombocytopenia but none of our patients had clinical findings of preeclampsia. Two patients did have mildly elevated liver enzymes, but as their blood pressure was within normal limits, the hepatic dysfunction was attributed to COVID-19 infection.

Moderate and severe thrombocytopenia in COVID-19 is considered a marker for disease severity but in our study, all the patients were asymptomatic on admission. Only one patient progressed to have a severe disease but she already had moderate thrombocytopenia on admission. It is possible that in these patients COVID-19 infection and gestational thrombocytopenia had a synergistic effect resulting in moderate or severe thrombocytopenia in asymptomatic patients. Abnormal coagulation function, including elevated D-dimer, has been demonstrated to be involved in the disease progression of COVID-19 [8]. In our third patient too, D-dimer levels were found to be elevated and she was started on anticoagulants for the same.

Regional anesthesia for cesarean section in COVID-19 positive patients has been recommended because of its obvious benefits. It can prevent the exacerbation of pulmonary complications of COVID-19 disease as well as avoid the high‑risk aerosol-generating procedure of intubation. Also, neuraxial anesthesia does not appear to alter the recovery of parturients from COVID-19 disease [9]. Regional anesthesia in the setting of reduced platelet count is also associated with an increased risk of epidural hematoma [10]. However, in pregnant women, a platelet count of 70,000 cells per mm^3^ has a low risk for spinal epidural hematoma, and lower levels should be considered in cases with a high risk for respiratory compromise with general anesthesia [11]. In our patients too, pre-operative platelet count was below 80,000 cells per mm^3^ but their cesarean section under spinal anesthesia was uneventful.

These cases highlight that moderate to severe thrombocytopenia may be associated with asymptomatic COVID-19 infection in pregnant women and a recent platelet count should be done before performing any neuraxial procedures in such patients. It also shows that thrombocytopenia in COVID-19 pregnant patients can be exaggerated due to factors like gestational thrombocytopenia and preeclampsia and obstetricians should be alert and closely monitor such patients especially pre- and post-operatively. It is recommended that, if required, donor blood products can be arranged before any operative procedure in these patients to minimize morbidity. A falling platelet count can also be an indicator for worsening of the disease and it reinforces the importance of close monitoring and follow-up of these patients.

## Conclusions

Thrombocytopenia in asymptomatic COVID-19 infection is usually mild but can be moderate to severe in COVID-19 infected pregnant patients, probably due to additional factors like gestational thrombocytopenia, preeclampsia, or HELLP syndrome. It is a challenge for the anesthetist and can lead to a dilemma to choose between the risks and benefits of neuraxial procedures. It also increases the risk of intraoperative and postoperative hemorrhage and can indicate the risk of progression to severe disease. It is therefore important that the obstetricians and anesthetists closely monitor pregnant patients with COVID-19 infection as they may have exaggerated findings when compared to the non-pregnant population. It is also advisable to check the coagulopathy parameters for these patients before any operative procedures.
